# Emergency treatment of symptomatic ureteral calculi: predictors of prolonged hospital stay

**DOI:** 10.1007/s11255-023-03749-0

**Published:** 2023-08-24

**Authors:** Roman Herout, Juliane Putz, Angelika Borkowetz, Christian Thomas, Sven Oehlschläger

**Affiliations:** Department of Urology, University Hospital Carl Gustav Carus, TU Dresden, Fetscherstraße 74, 01307 Dresden, Germany

**Keywords:** Urolithiasis, Emergency stone treatment, URS, SWL

## Abstract

**Purpose:**

To assess differences in the length of hospital stay (LOS) in patients who present emergently versus electively for a symptomatic ureteral stone and to explore underlying risk factors.

**Methods:**

Billing data were analyzed from patients with symptomatic ureteral calculi at our department from 2010 to 2021. Statistical analysis (*U t*est, logistic regression) was performed.

**Results:**

2274 patients (72% male, 28% female) with ureteral stones were analyzed (mean age of 52.9y). 1578 patients (69.4%) presented in an emergency setting and 696 patients (30.6%) electively. Arterial hypertension was seen in 31%, diabetes mellitus in 11% and hyperuricemia in 5% of the whole cohort. 46.5% of emergency patients were desobstructed (DJ/PCN), 35.4% underwent emergency ureteroscopy (URS), 13.4% had spontaneous passage (SP), and 4.8% underwent emergency shock wave lithotripsy (SWL). Of the electively treated patients, 58.6% underwent URS, 21.3% SWL, 18.5% DJ/PCN, and 1.6% had SP. Emergency stone treatment was associated with a significantly longer LOS when compared to primary desobstruction for patients admitted emergently. Also, LOS was significantly longer for each intervention of stone treatment in emergency patients vs. electively treated patients. Arterial hypertension was associated with a 1.8-fold increased risk of a hospital stay longer than 3 days, irrespective of hospital admission mode, whereas metabolic disorders did not influence LOS in this cohort.

**Conclusion:**

For emergency patients in contrast to the electively treated patients, the type of procedure had a significant impact on the length of hospital stay. Arterial hypertension is an independent significant risk factor for prolonged hospital stay.

## Introduction

Kidney stone disease is a common disorder with an increasing prevalence around the globe over the last decades [1, 2]. Recurrent stone formers are bearing an increased risk to develop chronic kidney disease (CKD) due to recurring blocking ureteral calculi [3–5]. All emergency procedures for symptomatic stones in the ureter are aimed at relieving the patient from pain, restoring urine flow in case of blocking calculi and, if possible, removal of the culprit(s) in a single session. Various factors, such as concomitant infection, stone size and location, patient preference, availability of a surgical method, and anesthesia, influence the choice of treatment for ureteral stones. In patients where an intervention is indicated, options range from primary desobstruction [double-J ureteral stent (DJ)/percutaneous nephrostomy tube (PCN)] to shock wave lithotripsy (SWL), ureteroscopy (URS), and very rarely antegrade percutaneous nephrolithotomy (PCNL) or open ureterolithotomy [6]. With its broad availability and excellent sensitivity and specificity, computed tomography (CT) has largely superseded the use of conventional kidney–ureter–bladder X-rays (KUB) and intravenous urography (IVU) as the diagnostic tool of choice for urolithiasis [7]. This trend was reinforced, after implementation of CT in the German and European guidelines in 2017 as a preferred diagnostic tool when stone disease is suspected [6, 8]. Over the last decades, endoscopic interventions for stone disease have become broadly available in many parts of the world, hence allowing for a patient-centered, individual approach to stone therapy. The impact of the mode of hospital admission, the type of intervention chosen to treat ureteral stones, as well as patients’ comorbidities with regards to length of hospital stay have not been thoroughly studied. Hence, we sought to describe current treatment patterns for ureteral stones in our center and explore the influence of the above-mentioned parameters on the length of hospital stay in this cohort.

## Materials and methods

The Institutional Review Board of the Technical University of Dresden approved the study protocol (Vote BO-EK-24012021).

We performed a billing data analysis of patients with symptomatic ureteral stones who were admitted to the Department of Urology at the University Clinic in Dresden from 2010 to 2021. The reimbursement of inpatient treatment is regulated by the diagnosis-related groups (DRG) in Germany. These DRGs are comprised of an ICD-10 (International Statistical Classification of Diseases and Related Health Problems) diagnosis code and an OPS (German Adaption of the International Classification of Procedures in Medicine) code for the performed intervention. Patients with indwelling ureteral stents or nephrostomy tubes at time of presentation (OPS codes 8–137.2, 8–137.1x, 8–138.1, Z97.8) as well as patients that underwent combined stone therapies (e.g., SWL and URS in the same hospital stay) were excluded from the analysis, since it was our aim to compare emergency procedures with elective surgery in a non-stented, naïve urinary tract. After exclusion of the above-mentioned cases, we identified consecutive patients with symptomatic ureteral calculi (ICD codes N13.2, N20.1, N20.2) between 2010 and 2021.

Patients in each group (emergency vs. elective) were then further subclassified in 4 subgroups according to therapeutic interventions: 1. insertion of DJ ureteral stent (DJ) or percutaneous nephrostomy tube (PCN) without URS or SWL (OPS 5–560.3, 8–137, 5–550.1), 2. ureteroscopy (OPS 5–562, 1–665 ± 8–137), 3. shock wave lithotripsy (OPS 8–110 ± 8–137), and 4. spontaneous passage of the ureteral calculus (no OPS code for the billed case). Also, patients who underwent computed tomography were identified by the specific OPS codes (3–206, 3–207, 3–225).

Comorbidity was assessed via the ICD codes for hypertension (I10), diabetes mellitus (E11), and hyperuricemia (E79.0). Effective length of hospital stay (LOS) was used as a comparative quality parameter.

Statistical analysis (Mann–Whitney *U *test and logistic regression) was performed using SPSS v.28.0.1.0 (IBM Corporation, Armonk, NY, USA).

## Results

**Fig. 1:**
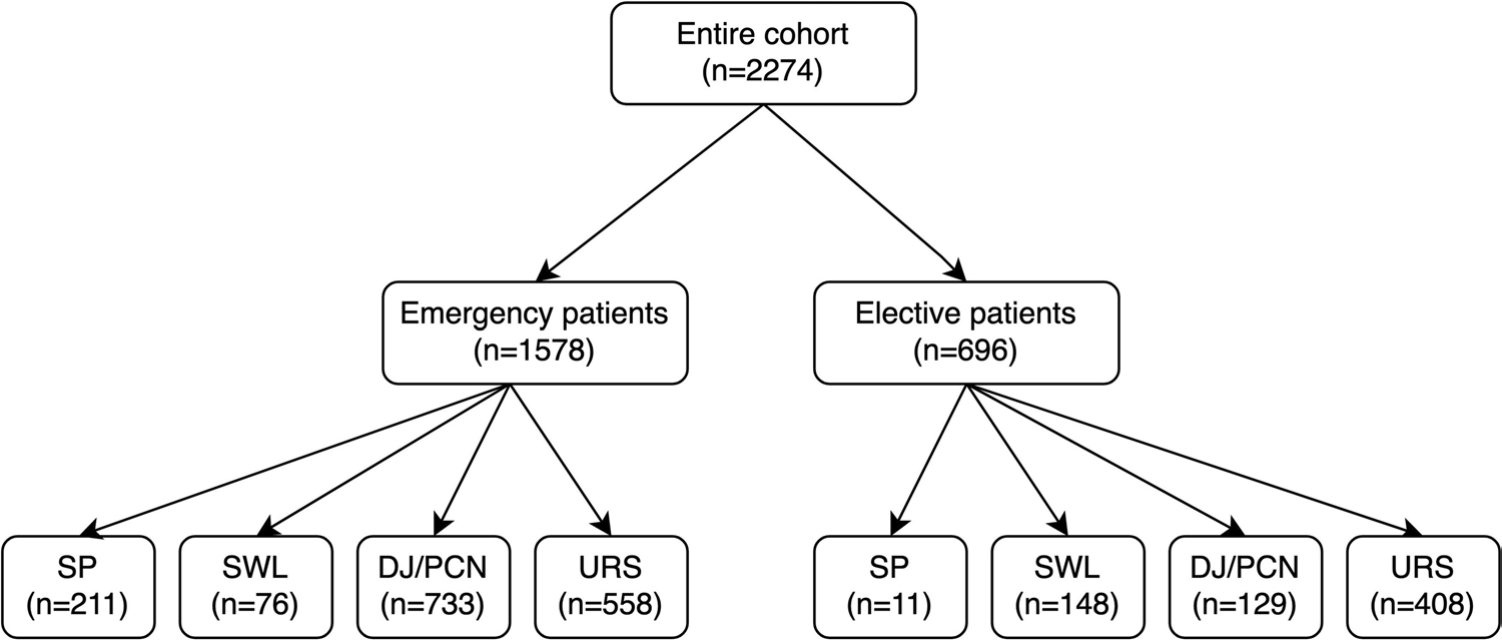
Flowchart of the group allocation of the study cohort. *SP*   spontaneous passage of the stone, *SWL* shock wave lithotripsy, *DJ/PCN* DJ ureteral stent or percutaneous nephrostomy, *URS* ureteroscopy

The study cohort consisted of 2274 consecutive patients, 1625 male (71.5%) and 649 female (38.5%), that were admitted to our department for the treatment of a single or multiple symptomatic ureteral stones. The mean age of patients was 52.9 years. As shown in Fig. 1, 1578 (69.4%) presented in an emergency setting and 696 patients (30.6%) were planned admissions for elective interventional therapy.

**Fig. 2:**
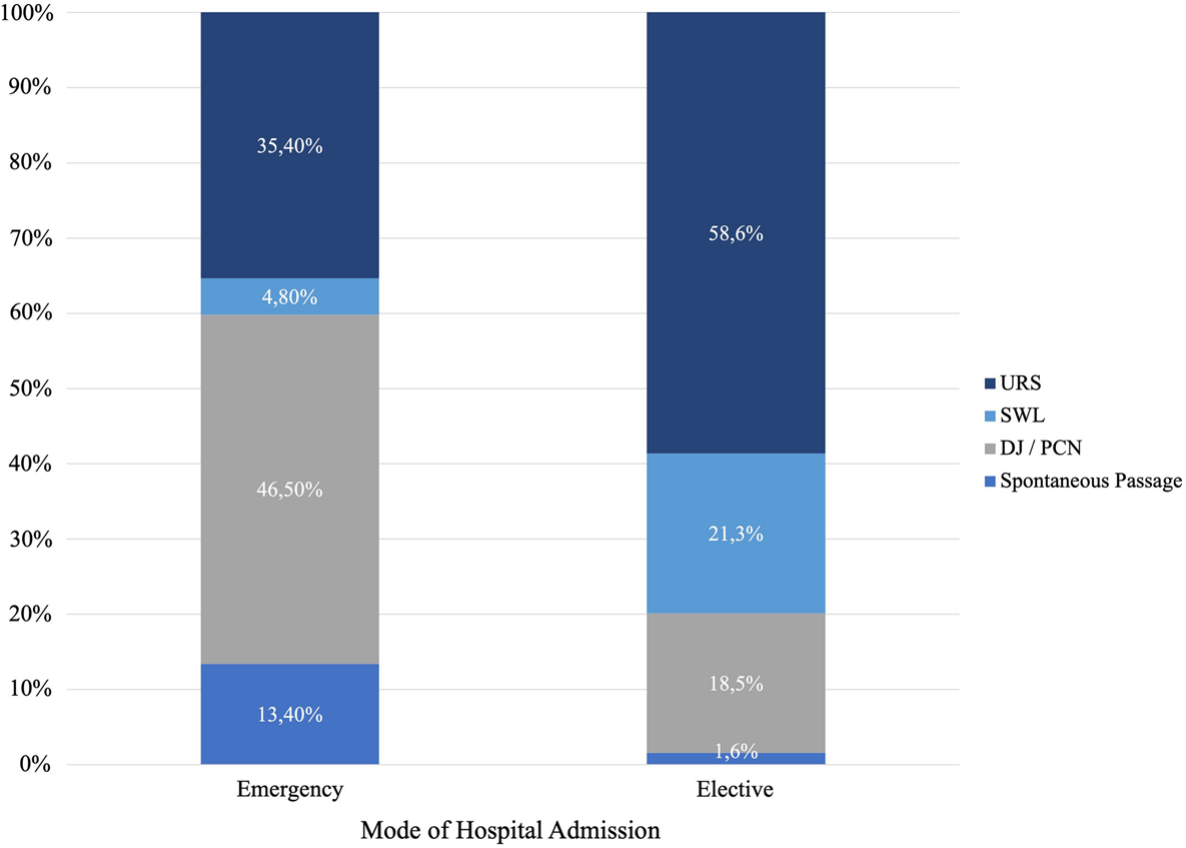
Distribution of therapeutic interventions according to hospital admission mode

The distribution of patients according to hospital admission mode and surgical therapy was as follows: 46.5% of patients that presented in an emergency setting received a DJ or PCN without further treatment of the stone during the same hospital stay. The most common therapeutic interventions in which the ureteral stone itself was treated was emergency URS (35.4%), followed by emergency SWL (4.8%). In 13.4% of patients, no intervention was necessary, as they passed the calculus spontaneously. In patients that underwent elective surgery, URS was the most common intervention (58.6%), followed by SWL (21.3%) and DJ/PCN insertion only (18.5%). Spontaneous passage of the ureteral stone occurred in 1.6% of patients (Fig. 2).

From 2010 to 2017, 221/968 patients (22.8%) had a CT scan, whereas from 2018 to 2021, 358/610 patients (58.6%) were diagnosed with computed tomography (*p* < 0.01). The spontaneous passage of the stone was significantly higher in patients who were admitted emergently and had a CT scan vs. emergency patients without CT scan (17.7% vs. 10.8%, *p* < 0.01). In a logistic regression model, the chances of a spontaneous stone passage were significantly improved by having an emergency CT scan of the calculus (odds ratio = 2.4).

Arterial hypertension was seen in 711 of the 2274 patients (31.2% of the whole cohort), diabetes mellitus in 247/2274 (10.8%), and hyperuricemia in 112/2274 (4.9%). Patients with hypertension had a 1.8-fold increased risk of a hospital stay longer than 3 days when compared to patients without hypertension. No statistical significance regarding the LOS was seen for patients with diabetes and hyperuricemia.

In Table 1, the mean lengths of stay for the emergency and elective patients regarding the chosen interventions are shown. We observed a significantly shorter mean length of hospital stay for patients who were admitted for elective surgery for each intervention (DJ/PCN only, SWL, or URS) when compared to emergency patients. In patients that had a spontaneous passage of the ureteral stone, no statistical difference with regards to hospital stay could be detected.

**Table 1:** Length of hospital stay with regard to intervention

Intervention	Emergency patients LOS (d) *n* = 1578	Elective patients LOS (d) *n* = 696	Statistical significance (Mann–Whitney *U *test)
DJ/PCN only (OPS: 8–137.0x, 5–560, 5–550.1)	3.05 ± 1.85 d (*n* = 733)*/^†^	2.40 ± 1.78 d (*n* = 129)	*p* < 0.01
SWL (OPS: 8–110 ± 5–560.3, 8–137.0x, 5–550.1)	3.89 ± 2.39 d (*n* = 76)*	2.61 ± 2.08 d (*n* = 148)	*p* < 0.01
URS (OPS: 5–562 ± 5–560.3, 8–137.0x, 5–550.1)	3.30 ± 1.76 d (*n* = 558)^†^	2.70 ± 1.23 d (*n* = 408)	*p* < 0.01
Spontaneous passage of the stone (no associated OPS code)	1.74 ± 0.99 d (*n* = 211)	1.45 ± 0.69 d (*n* = 11)	*p* = 0.360

Mann–Whitney *U *test: */^†^ = *p* < 0.01

In our statistical model, all surgical interventions (DJ/PCN insertion, URS, SWL) of emergency patients were associated with an increased risk of a hospital stay > 3d (ORs: DJ/PCN = 7, URS = 9.4, SWL = 15.3). This trend could not be observed for the same analysis in the elective patient cohort, i.e., the elective patients are only at risk (OR 1.8) when they suffer from arterial hypertension.

## Discussion

In this study, we present contemporary data on the treatment of symptomatic ureteral stones and analyzed factors that impact the length of hospital stay in emergently admitted patients versus patient that underwent elective surgery.

The length of hospital stay as an outcome measure for quality of care is well established and longer hospital stays correlate to worse clinical outcomes for patients with higher morbidity and mortality [9, 10]. Hanau et al. have shown that comorbidity prolongs the hospital stay in patients undergoing flexible ureteroscopy [11]. Similarly, this association between comorbidity and prolonged hospital stays has been demonstrated for other urological and surgical procedures [12, 13].

Seklehner et al. published Medicare data from the United States including almost 300,000 patients that underwent treatment of ureteral stones, where they described an increase in the use of URS at the expense of SWL (64.4% underwent URS vs. 29.6% underwent SWL) [14]. These data were comparable with our group of electively admitted patients with URS (58.6%) and SWL (21.3%) treatments. In the emergency group, the URS was performed in only 35.4% and the SWL in 4.8%.

The type of intervention had a significant impact on the length of hospital stay in patients that were admitted emergently, i.e., emergency patients that received a DJ or PCN had a significantly shorter LOS when compared to patients who underwent definitive treatment in the emergency setting (URS or SWL). In patients that were admitted electively, we did not observe a significant difference in LOS regarding the type of chosen intervention. This finding implies that somehow emergency patients could be inherently at higher risk for longer hospitals stays due to complicating factors. This is especially true for the “complex” patient with a high stone burden, a stone in an unfavorable location (mid-ureter, proximal ureter), anatomical factors (narrow ureteral orifice, narrow ureter), or concomitant urinary tract infection [15]. The reported incidence of urinary tract infections (UTI) in patients presenting emergently with obstructive ureteral stones varies in the literature between 8 and 22% [16, 17]. According to our institutional workflow, patients with signs of UTI and/or fever undergo emergency decompression rather than emergency URS or SWL and the former had a significantly shorter hospital stay when compared to the other groups.

There is increasing evidence from single-center, non-randomized, retrospective series that emergency URS is a feasible option in patients without complicating factors, such as UTI, fever, or concerns regarding anesthesia. However, in the reported literature, success rates of emergency URS tend to be higher in younger patients with lower American Association of Anesthesiologists (ASA) scores and smaller stones located in the distal ureter [18, 19].

Arterial hypertension was the most common comorbidity in our cohort, observed in about a third of patients. This disease is often associated with other comorbidities, such as coronary artery disease, stroke, chronic kidney disease, heart failure, and chronic obstructive pulmonary disease [20]. During surgery, hypertensive patients are regarded to be at an increased risk for organ damage, especially acute kidney injury (AKI), due to hypoperfusion during hypotensive episodes which affects the perioperative outcome [21]. In a study of 7740 surgical patients, with 957 of them undergoing urological surgery, hypertension was found to be an independent predictor of a postoperative cardiac adverse event, defined as cardiac arrest, myocardial infarction, or new cardiac dysrhythmia, with a hazard ratio of 1.7 [22]. In our cohort, patients with arterial hypertension had a 1.8-fold increased risk of having a prolonged hospital stay (longer than 3 days), irrespective of hospital admission mode.

Today, computed tomography (CT) is broadly available, and by virtue of its high sensitivity and specificity, it has become the imaging modality of choice to diagnose stones in the upper urinary tract [7, 19]. In 2017, the European (EAU) and German (DGU) guideline panels gave a recommendation for CT as first-line imaging modality in acute flank pain. We could demonstrate a significant increase in patients receiving a CT scan when comparing the years 2010–2017 vs. 2018–2021 with 22.8 and 58.6% of patients undergoing a CT scan, respectively. CT allows for three-dimensional reconstruction of the stone with volume rendering, exact determination of stone location and gives a hint regarding stone composition via measuring density in Hounsfield units. In our logistic regression model, the chance for spontaneous stone passage for patients with CT scans was significantly improved (odds ratio = 2.4). We hypothesize that due to higher diagnostic accuracy when compared to KUB and IVP, patients with CT scans have a greater chance, if possible, of being managed conservatively with spontaneous stone passage than patients without CT scans. Hence, the information about the exact stone size and location as well as possible mineralogical composition enables treating physicians to either start/continue medical expulsion therapy (MET), MET plus alkalization of urine in suspected uric acid stones, emergency decompression in cases with complicating factors, or emergency URS or SWL according to the current AUA and EAU guidelines [23, 24].

### Limitations

We cannot provide details on exact stone locations (lower, mid, or upper ureter) and sizes, which are substantial in the complex treatment process for urolithiasis. Due to the German health care system reimbursement regulated via DRG, we cannot exclude that factors that are inherent to the complex system might have influenced the LOS. Additionally, group sizes to analyze the impact of comorbidities were small for patients with diabetes (11% of the entire cohort) and hyperuricemia (5%).

## Conclusion

The type of procedure had a significant impact on the length of hospital stay for emergency patients and no influence was seen in the electively treated patients. Arterial hypertension is an independent and significant risk factor for prolonged hospital stay in this patient cohort. Awareness of hypertensive patients being at risk for acute kidney injury (AKI) as well as optimization of antihypertensive therapy between emergency decompression and scheduled definitive stone therapy and optimal fluid management during surgery might lead to improved outcomes and reduce the risk of AKI and a prolonged hospital stay. A staged approach with primary desobstruction and delayed definitive treatment seems justified in patients with comorbidity and complicating factors of primary stone treatment in complex stone disease.

## Data Availability

The data presented in this study are available upon reasonable request from the corresponding author via roman.herout@uniklinikum-dresden.de.
